# Supplementary material for: Determination of Binding Affinity of Antibodies to HIV-1 Recombinant Envelope Glycoproteins, Pseudoviruses, Infectious Molecular Clones, and Cell-Expressed Trimeric gp160 Using Microscale Thermophoresis
Source: Cells. 2023 Dec 22;13(1):33. doi: 10.3390/cells13010033 (PMC10778174; doi:10.3390/cells13010033)
Supplement: Supplementary file 1 [file cells-13-00033-s001.zip › cells-2674609-supplementary.pdf]

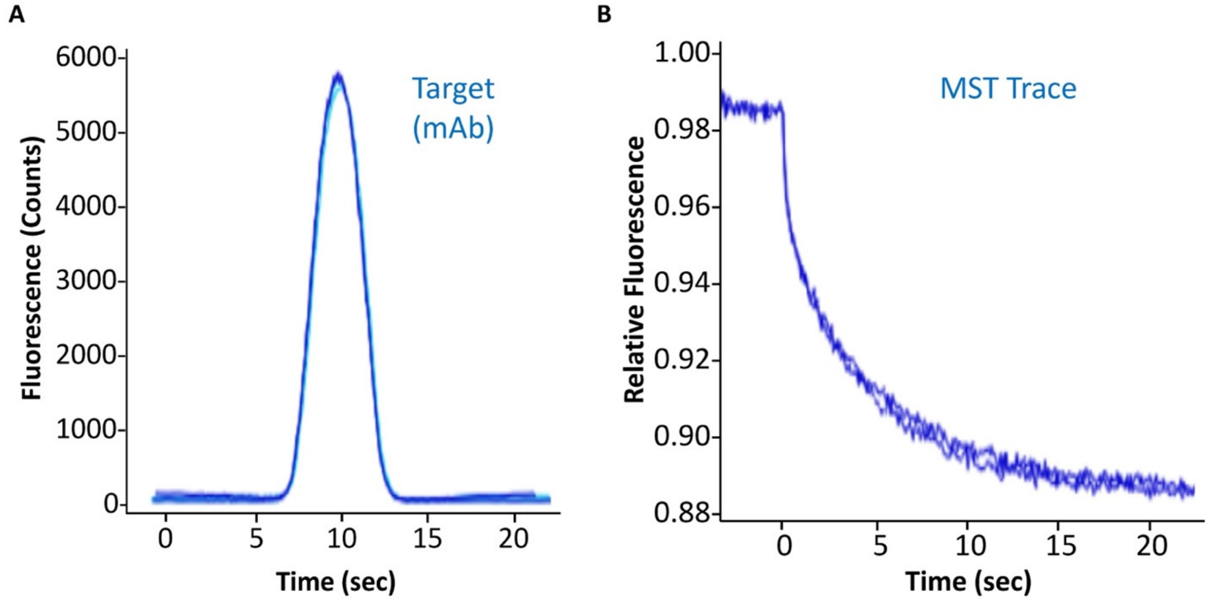

Figure S1. Quality check of the fluorescently labeled monoclonal antibody (mAb), DH827. (A) A capillary scan representing the fluorescence counts on the Y-axis, and time (seconds) on the X-axis. Labeled DH827 mAb represents normal capillary fluorescence shape and fluorescence intensity without variation and adsorption. (B) Normal microscale thermophoresis (MST) trace showing relative fluorescence on the Y-axis, and time (seconds) on the X-axis, and the relative labeled DH827 mAb curve without any aggregation.

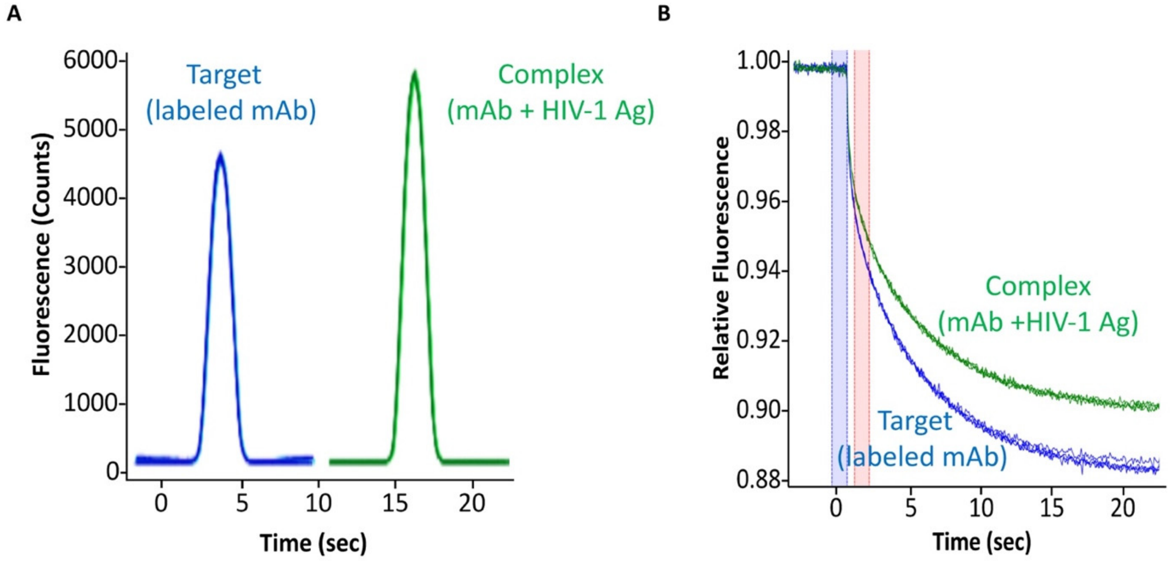

Figure S2. Binding curve from a Microscale Thermophoresis (MST) experiment showing the interaction between labeled monoclonal antibody (mAb), DH827 (50 nM) and acute subtype C gp145 (C6980V0C72, 75 nM) HIV-1 Env protein. (A) MST capillary scan showing the fluorescence counts on the Y-axis and time (seconds) on the X-axis. The capillary scan represents normal

fluorescence intensity of the target molecule (labeled DH827 mAb; blue color), and complex molecule (labeled mAb DH827+ acute C gp145 Env protein; green color) without any fluorescence variation, adsorption, aggregation, and ligand (acute C gp145 Env protein) induced photobleaching changes. (B) MST output using the M.O. affinity analysis software V2.1 representing the fluorescence change of gp145-DH827 mAb complex at different time points after the start of the IR laser as per the change of movement of the complex molecules due to thermophoretic motion differences. The smooth MST dose response curve confirms the binding interaction between the fluorescently labeled DH827 mAb-gp145 complex.

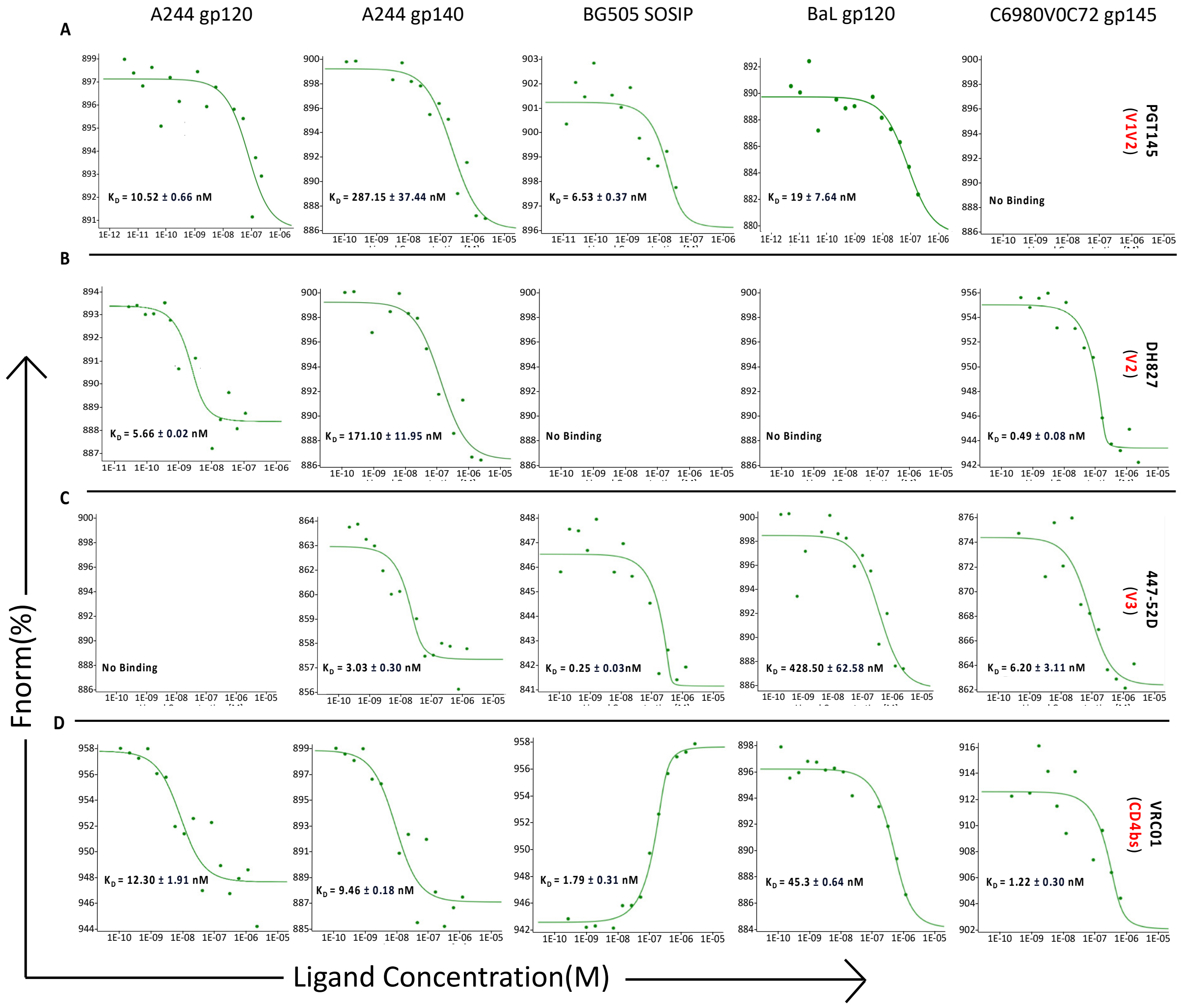

Figure S3. Representative microscale thermophoresis (MST) traces of binding affinities (K_D) of different monoclonal antibodies (mAbs) with monomers and trimers of HIV-1 envelope proteins. Experiments were performed at room temperature for 30 minutes. The graphs are presented as Fnorm [%] vs ligand concentration (M). MST traces showing binding curves and affinities of mAbs (A) PGT145 (25 nM), (B) DH827 (50 nM), (C) 447-52D (5-30 nM), and (D) VRC01 (12.5-15 nM),

respectively to HIV-1 Env monomer (A244 and BaL gp120), and trimer (A244 gp140, BG505 SOSIP, and C6980V0C72 gp145) proteins (75-300 nM final concentration).

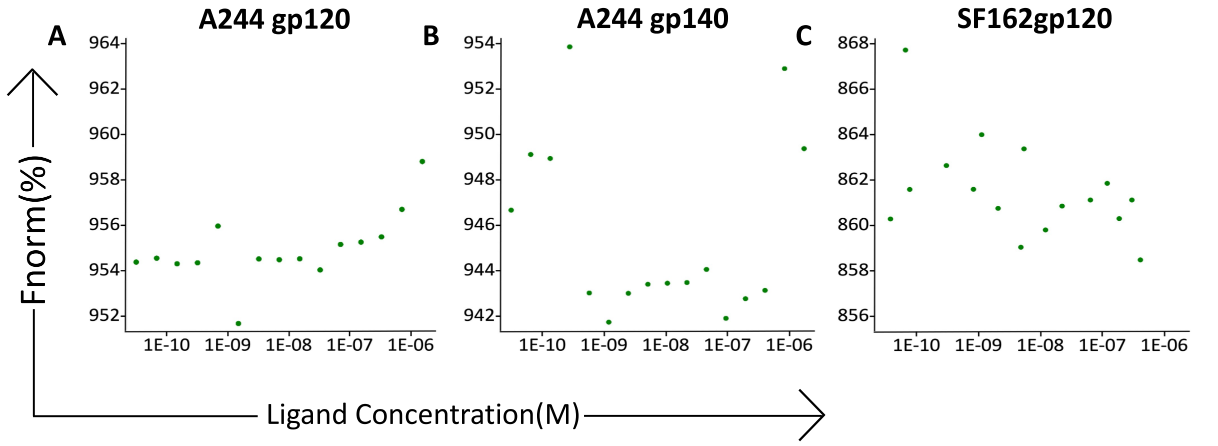

Figure S4. Microscale thermophoresis (MST) traces of fluorescently labeled respiratory syncytial virus (RSV) mAb Synagis with HIV Env proteins. No binding was observed with Synagis and HIV-1 Env proteins (A) A244 gp120, (B) A244 gp140, and (C) SF162 gp120. The graphs are represented as Fnorm [%] vs ligand concentration (M). The experiments were performed at room temperature for 30 minutes.

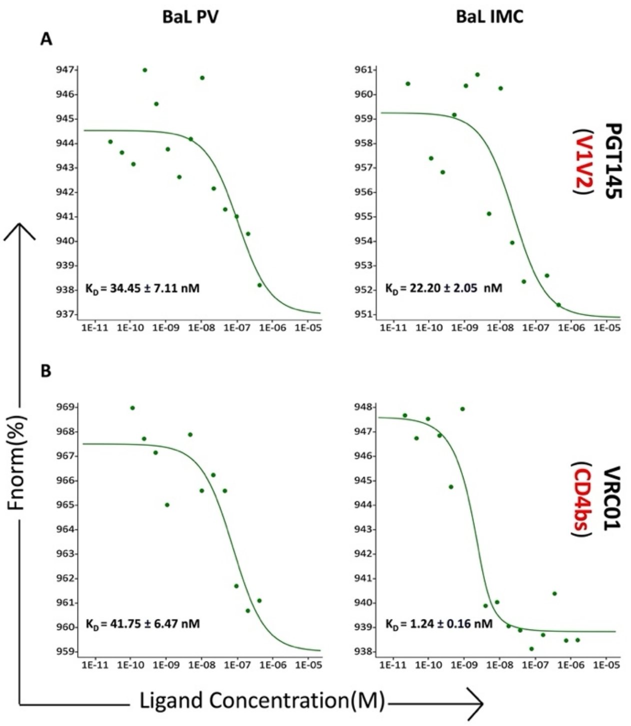

Figure S5. Representative microscale thermophoresis (MST) traces of binding affinities (K_D) of bNAbs to BaL Pseudovirus (PV) and Infectious Molecular Clone (IMC). To determine the binding affinity, capillaries were loaded with 12.5 nM each of fluorescently labeled (A) PGT145 or (B) VRC01 mAbs along with serially diluted (1:1) BaL PV or IMC (0. 1 mM and 0.18 µM respectively, based on the p24 concentration) in a final volume of 10 µL. Binding experiments were performed at room temperature for 30 minutes. The graphs are represented as Fnorm [%] vs ligand concentration (M).

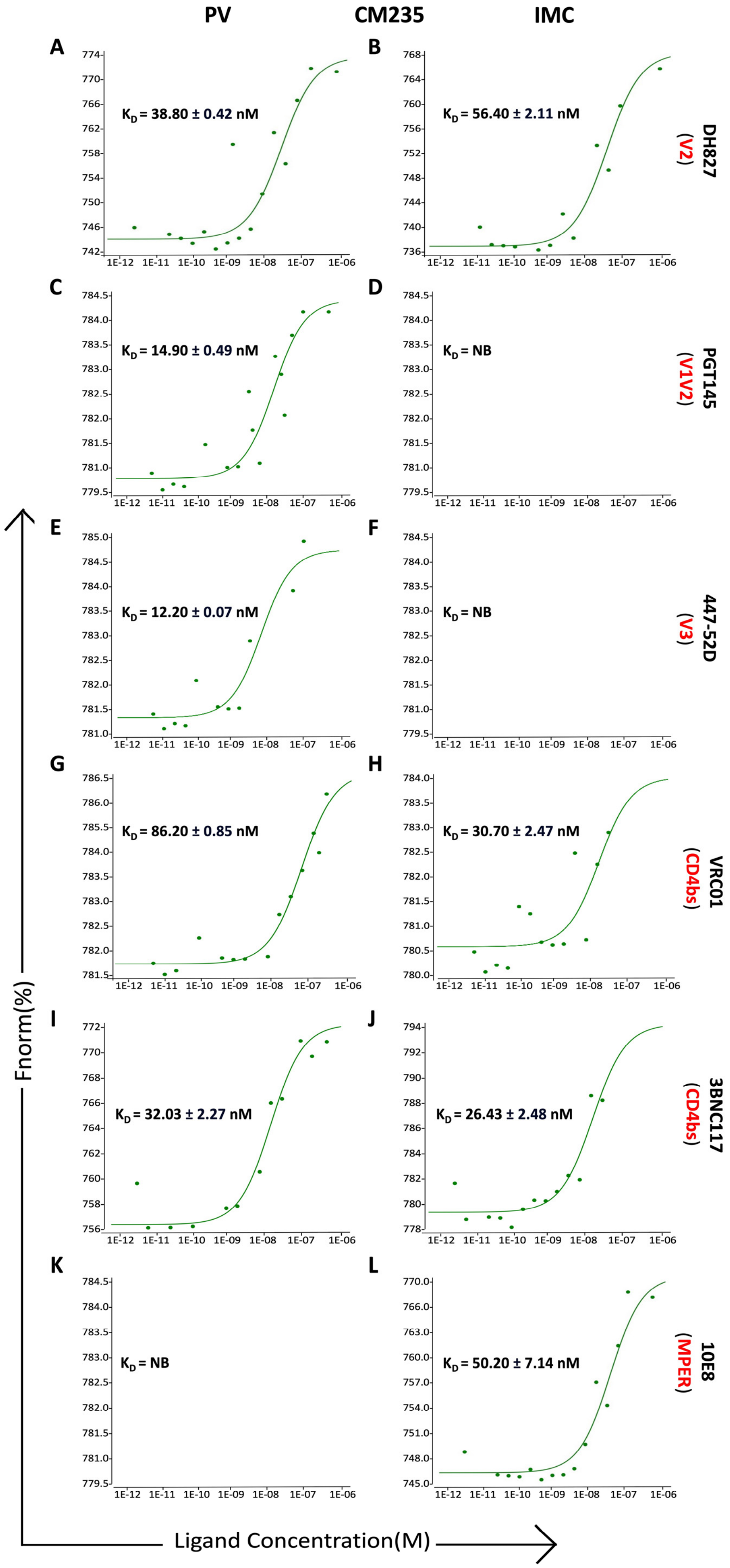

PV
CM235
IMC
A
B
C
D
E
F
G
H
I
J
K
L
K_D = 38.80 ± 0.42 nM
K_D = 56.40 ± 2.11 nM
DH827 (V2)
K_D = 14.90 ± 0.49 nM
K_D = NB
PGT145 (V1V2)
K_D = 12.20 ± 0.07 nM
K_D = NB
447-52D (V3)
K_D = 86.20 ± 0.85 nM
K_D = 30.70 ± 2.47 nM
VRC01 (CD4bs)
K_D = 32.03 ± 2.27 nM
K_D = 26.43 ± 2.48 nM
3BNC117 (CD4bs)
K_D = NB
K_D = 50.20 ± 7.14 nM
10E8 (MPER)
Fnorm(%)
Ligand Concentration(M)

Figure S6. Representative microscale thermophoresis (MST) traces of binding affinities (K_D) of monoclonal antibodies (mAbs) to CM235 Pseudovirus (PV) and Infectious Molecular Clone (IMC). The labeled mAbs were used at the concentration of 12.5 nM and the experiments were performed at room temperature for 30 minutes. To determine the binding affinity, capillaries were loaded with fluorescently labeled mAbs and serially diluted (1:1) CM235 PV or CM235 IMC (final concentration of 0. 1 mM and 0.18 µM respectively, based on the p24 concentration) in a final volume of 10 µL. MST traces of binding affinities of mAbs (A, B) DH827, (C, D) PGT145, (E, F) 447-52D, (G, H) VRC01, (I, J) 3BNC117, and (K, L) 10E8 respectively to CM235 PV and IMC. PGT145 and 447-52D did not bind to CM235 IMC. Similarly, no binding was observed between 10E8 and CM235 PV. The graphs are represented as Fnorm [%] vs ligand concentration (M).

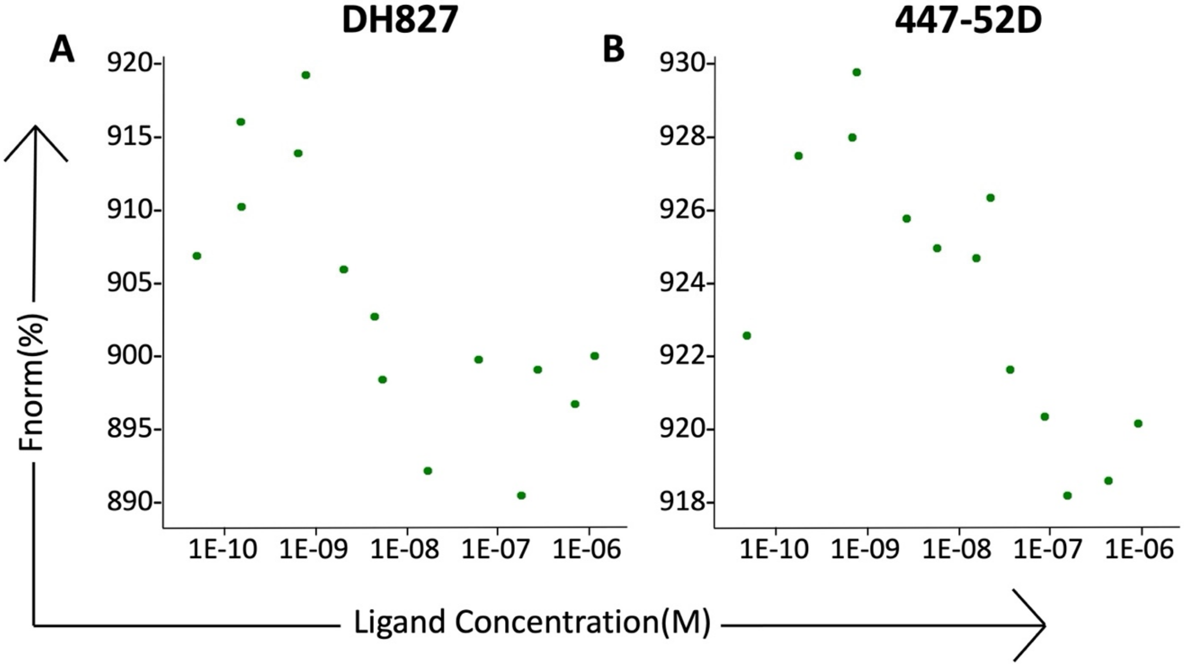

Figure S7. Microscale thermophoresis (MST) traces of monoclonal antibodies (mAbs) with the parental cell line (CEM.NKR.CCR5). To determine the binding affinity, capillaries were loaded with fluorescently labeled mAbs (A) DH827 (50 nM) and (B) 447-52D (20 nM) and serially diluted (1:1) parental cell line (CEM.NKR.CCR5) that does not express the HIV-1 env protein at a starting concentration of $5x10^6$ cells/mL in a final volume of 10 µL. The binding experiments were performed at room temperature for 30 minutes. The graphs are represented as Fnorm [%] vs ligand concentration (M). No binding was observed to the parental cell lines with either mAb.

Table S1. Comparative binding affinities of fluorescently labeled monoclonal antibodies (mAbs) to monomer (A244 gp120), and trimer (BG505-SOSIP) HIV-1 proteins determined by Microscale thermophoresis (MST) and previously published data determined by SPR, Octet, or ITC.

	**A244 gp120**		**BG505-SOSIP**		
	**Binding Affinities (KD nM)**		**Binding Affinities (KD nM)**		
**Monoclonal Antibodies (mAbs)**	**MST**	**SPR/Octet**	**MST**	**SPR/Octet/ITC**	**References (PMID)**
PGT145	11	NA	7	2.57	32056466
				2.9	24884783
				5.7	25422458
PGDM1400	NB	NA	1	5.5	25422458
PG9	208	42.1*, 81.6	93	NA	23313589
				75	24884783
		183		NA	23175357
		NB		NA	29698406
				36	23426631
				2.1	32056466
DH827	6		NB	NA	
CH58	20	1.0*, 0.12	NB		23313589
				NB	32056466
		1.58			29698406
CH59	21	2.2*, 0.48	NB	NA	23313589
		0.90			29698406
447-52D	NB	NA	0.3	NA	
2G12	6		4	1.3	24884783
				$<1.0 \times 10^{-3}$	32056466
				16	24068931
VRC01	12	15.7	2	NA	23175357
				2.1×10^{-3}	32056466
				0.72	24884783
				0.46	28972148
		55.5			29698406
3BNC117	35		7	0.19	28972148
		NA			

* A244 gp120, 11th amino acid deleted

NB: No binding

NA: Not Available
